# Understanding the impact of COVID-19 on women’s access to and experiences of contraceptive services in England: a qualitative study

**DOI:** 10.1136/bmjsrh-2023-202206

**Published:** 2024-03-19

**Authors:** Lauren McMillan, Erica Gadsby, Rebecca Howell, Michael Ussher, Kate Hunt, Allison Ford

**Affiliations:** 1 Institute for Social Marketing and Health, University of Stirling, Stirling, UK; 2 Population Health Research Institute, St George's University of London, London, UK

**Keywords:** COVID-19, contraception behavior, Health Services Accessibility, Health Services Research, Health Policy, qualitative research

## Abstract

**Background:**

The COVID-19 pandemic response prompted rapid changes to how contraceptive services were delivered in England. Our aim was to examine women’s experiences of accessing contraceptive services since March 2020 and to understand any inequalities of access.

**Methods:**

We conducted telephone interviews with 31 women aged 17–54 years who had accessed contraceptive services in England since March 2020. The sample was skewed to include participants with lower educational attainment and higher deprivation. Interview transcripts were thematically analysed using inductive and deductive approaches.

**Results:**

Few differences were found regarding educational attainment. Participants using contraceptive injections (all living in areas in the most deprived quintile) reported the greatest access challenges. Some switched method or stopped using contraception as a result. More general barriers reported by participants included service closures, unclear booking processes, and lack of appointment availability. Many participants welcomed the flexibility and convenience of remote contraceptive services. However, telephone appointments posed challenges for those at school or living with parents, and some described them as rushed and inconducive to asking questions or raising concerns. Those accessing contraception for the first time or nearing menopause felt they were unable to access sufficient support and guidance during the pandemic. Some participants voiced concerns around the lasting effects of COVID-19 on appointment availability and inadequate service delivery.

**Conclusions:**

Women’s experiences of accessing contraceptive services in England since March 2020 are diverse. While remote services were suitable for some, COVID-19 restrictions unequally impacted women depending on their method of contraception and life stage.

**WHAT IS ALREADY KNOWN ON THIS TOPIC**

- COVID-19 impacted the delivery of contraceptive services. Remote service delivery was adopted to overcome access barriers. Face-to-face restrictions led to a drop in prescribing rates.

**WHAT THIS STUDY ADDS**

- Women using contraceptive injections reported the greatest access barriers. Suitability of remote service delivery varied depending on circumstance with life stage as an indicator.

**HOW THIS STUDY MIGHT AFFECT RESEARCH, PRACTICE OR POLICY**

- Policy must consider how individual needs are met and in-depth support provided if a more remote model of service delivery becomes routine practice longer term.

## Introduction

 Accessible contraception is essential to sexual and reproductive health and well-being^1^ and prevents unintended pregnancies.^2^ The COVID-19 pandemic impacted access to contraceptive services globally.^3^ In developing countries, medication shortages and supply chain issues created barriers to access, while developed countries faced staffing and appointment cutbacks.^4^ In England, face-to-face appointments for contraceptive services were restricted and sexual health clinics (SHCs) closed.^5^ These disruptions led to a decrease in prescribing rates across England for combined oral contraception, contraceptive injections and emergency contraception.^6^ Prescriptions of long-acting reversible contraception (LARC) methods reduced by 77% over the first 3 months of lockdown^7^ and LARC prescribing rates had not recovered to pre-pandemic levels by December 2022.^8^ A UK cohort study found that women who conceived between April and December 2020 reported higher proportions of unintended pregnancies than those who conceived before lockdown measures came into force in March 2020.^9^

To overcome access issues caused by COVID-19, the Faculty of Sexual & Reproductive Healthcare deemed it essential that women be able to access contraception, and developed UK-wide guidance for contraceptive services during the pandemic.^10^ The guidance recommended a “digital first” approach to service delivery and pharmacy provision of the progestogen-only pill and contraceptive injections.^11 12^ Although these changes were seen as progressive and were widely accepted,^3 13 14^ many women encountered difficulties in accessing their preferred contraceptive.^9 15 16^ An interview study found that navigating remote services required tenacity, and some expressed concerns around privacy and the quality of remote interactions.^15^ Furthermore, vulnerable populations were disproportionately affected. Younger women were more likely to report access challenges, amplified by inconsistent and confusing advice,^17 18^ while the rapid implementation of remote services deepened concerns that those from disadvantaged socioeconomic backgrounds would face digital exclusion.^19^

For context, sexual health services in England are commissioned at a local level to meet the needs of the population, meaning there is considerable regional variation in how contraceptive services are provided. They vary from distinct general practitioner (GP), SHC, and pharmacy provision to fully integrated models within the community.^20^ In view of the shifting landscape around contraception provision in England, there is a need to advance in-depth understanding of how women experience contraceptive services. This study aimed to examine experiences of accessing contraceptive services post-March 2020, both for new users of contraception and those with prior experience, and to understand inequalities of access.

## Methods

### Study design

A qualitative study using semi-structured telephone interviews was conducted to explore women’s experiences of accessing contraceptive services since March 2020. The study followed Consolidated Criteria for Reporting Qualitative Research.^21^ Telephone interviews were selected for effective data-gathering across sample criteria, while minimising participant burden and offering anonymity while discussing potentially sensitive topics. Eligible participants were aged 16 to 54 years, had accessed contraception since March 2020, and were living in England. The study received approval from Stirling University General University Ethics Panel (GUEP 10259).

### Participants

Participants were recruited through a General Data Protection Regulation (GDPR)-compliant market research agency. To ensure a spread of demographic characteristics, minimum quotas were placed on: age group; whether women had only accessed contraceptive services since March 2020 (new users) or had accessed services before and after March 2020; educational attainment level; and Index of Multiple Deprivation (IMD) quintile score (skewed towards those with lower educational attainment and IMD1). Participants were approached by the market research agency via an existing panel of adults (aged 16+ years) who had expressed interest in research. Interested participants completed a short online recruitment questionnaire to assess eligibility. Those who met the criteria and agreed to participate were invited by the agency to complete an online consent form. Telephone interviews were arranged once informed consent was obtained.

### Data collection

Thirty-one semi-structured telephone interviews were conducted by AF, a female research fellow with substantial experience in qualitative research and two female research assistants (LM, RH) between November 2022 and January 2023. Participants knew that AF, LM and RH were researchers working on the study. A semi-structured topic guide was used to explore participants’ experiences of accessing contraceptive services, perceived changes in access since March 2020, barriers and facilitators, knowledge of services, and attitudes towards different access modes. The guide was informed by the Theoretical Domains Framework (TDF),^22^ which defines 14 domains of health behaviour. We used the TDF to facilitate a comprehensive assessment of the determinants of participants’ contraceptive behaviours during the pandemic, and to identify barriers and facilitators to these behaviours. Interviews were digitally audio-recorded, and lasted 38 to 86 min, except for one interview which lasted 7 min due to a lost connection. Participants received £40 as a reward for their time.

### Data analysis

The audio-recordings were professionally transcribed verbatim. AF, LM and RH checked all transcripts for accuracy and removed identifiable information before analysis. The data were thematically analysed^23^ using both deductive (informed by the topic guide) and inductive (derived from participants’ accounts) approaches. Key themes and issues were initially identified through familiarisation and a draft coding framework was developed by AF and LM, and via discussions with the research team. The coding framework was tested independently by AF and LM who double-coded three transcripts, before discussing and refining the framework. Using NVivo12, transcripts were coded by AF, LM, EG and RH. AF checked a sample of transcripts to ensure consistency of coding between researchers. Once transcripts had been coded, LM summarised the data and identified salient differences and similarities across cases. Themes were initially interpreted by LM and refined and agreed through discussion with AF, KH and EG and key quotes selected. To minimise any bias that might be introduced by their beliefs, the authors reflected on the potential impacts that their shared gender identity (cisgender women) and own lived experience might have on the findings.

### Patient and public involvement statement

The interview topic guide was informed by two online public consultation groups facilitated by AF and LM in November 2022 with nine women living in England, split by age (18–24 and 25–54 years). Participants responded to an initial set of questions developed for use in the topic guide and were encouraged to speak openly about their contraceptive experiences during the pandemic. This provided a preliminary understanding of the most salient issues, and insight into topic areas that the particpants suggested prioritising. Vital information from the groups around a lack of choice of contraceptive methods and poor communication of the services available during the pandemic helped shape the topic guide.

## Results

Tables1  2 demonstrate that the 31 interviewees comprised a diverse group in terms of contraceptive methods used, types of services accessed, and the mode by which services/methods had been accessed. Most of the particpants had used multiple methods or accessed more than one service.

**Table 1:** Sample characteristics

Characteristic	n
Age (years)	
17–29	17
30–39	5
40–54	9
Ethnicity	
White	16
Black African	2
Mixed White and Black African	1
Asian	10
Other ethnic group	1
Prefer not to say	1
Locality	
Urban	16
Suburban	11
Rural	4
Region	
East Midlands	2
East of England	0
London	11
North East	0
North West	4
South East	1
South West	6
West Midlands	3
Yorkshire and The Humber	4
Educational attainment	
A-levels or equivalent and above	15
GCSEs or equivalent or below	16
IMD quintile*	
1	15
2	5
3	9
4	2
5	0
New user of contraceptive services since March 2020	
Yes	8
No	23
All contraceptive methods used since March 2020	
Barrier methods	16
Contraceptive pill (combined or progestogen only)	12
Emergency oral contraception	12
Contraceptive implant	8
Intrauterine system	7
Contraceptive injections	4
Calendar method	4
Withdrawal	4
Contraceptive patch	2
Fertility awareness apps	2
Intrauterine device	1
Emergency intrauterine device	1
Vaginal ring	1
Diaphragm plus spermicides	1
All services usually accessed	
General practice	20
Sexual health/family planning clinic	12
Pharmacy	16
Online pharmacy	2
Other online source	2
Retail outlet	3
School/college/university	1
NHS postnatal provision	2
All modes of access since March 2020	
Face-to-face	12
Telephone	14
Online	8

* 1=most deprived, 5=least deprived.

GCSEGeneral Certificate of Secondary EducationIMDIndex of Multiple DeprivationNHSNational Health Service

**Table 2:** Individual sample characteristics

Participant ID	Age (years)	Ethnicity	Locality	IMD quintile*	Educational attainment	New user since March 2020	Mode of access used
P1	30–39	White British	Rural	4	Bachelor’s degree or equivalent	Yes	Face-to-face
P2	40–54	White British	Rural	3	Bachelor’s degree or equivalent	Yes	Telephone
P3	17–29	Any other Asian background	Urban	1	A-levels or equivalent	No	Online
P4	17–29	Any other White background	Suburban	3	Postgraduate degree or equivalent	Yes	Face-to-face
P5	17–29	African	Urban	1	A-levels or equivalent	Yes	Online
P6	30–39	Any other ethnic group	Suburban	1	A-levels or equivalent	Yes	Online
P7	17–29	White British	Urban	1	A-levels or equivalent	No	Face-to-face
P8	17–29	Indian	Urban	3	A-levels or equivalent	Yes	Telephone
P9	17–29	Pakistani	Urban	1	10 GCSEs or equivalent	Yes	Online
P10	17–29	Chinese	Urban	3	A-levels or equivalent	No	Face-to-face
P11	17–29	African	Suburban	3	Postgraduate degree or equivalent	No	Face-to-face
P12	17–29	Indian	Urban	1	No qualifications	No	Telephone
P13	40–54	Pakistani	Urban	3	Postgraduate degree or equivalent	Yes	Face-to-face
P14	17–29	White British	Rural	1	10 GCSEs or equivalent	Yes	Telephone
P15	17–29	White British	Rural	1	10 GCSEs or equivalent	Yes	Telephone
P16	17–29	Any other Asian background	Suburban	1	7–9 GCSEs or equivalent	No	Telephone
P17	30–39	Pakistani	Urban	1	<5 GCSEs or equivalent	Yes	Online
P18	40–54	White European	Suburban	1	No qualifications	Yes	Face-to-face
P19	40–54	Any other White background	Urban	3	Bachelor’s degree or equivalent	Yes	Telephone
P20	17–29	White British	Suburban	1	7–9 GCSEs or equivalent	Yes	Face-to-face, telephone
P21	17–29	Prefer not to say	Urban	2	7–9 GCSEs or equivalent	No	Face-to-face
P22	40–54	White British	Suburban	2	<5 GCSES or equivalent	Yes	Face-to-face, online
P23	40–54	Pakistani	Suburban	1	<5 GCSES or equivalent	Yes	Face-to-face, telephone
P24	17–29	White British	Urban	4	7–9 GCSEs or equivalent	No	Telephone
P25	40–54	White British	Suburban	3	Postgraduate degree or equivalent	Yes	Online
P26	30–39	Any other White background	Urban	2	<5 GCSEs or equivalent	Yes	Telephone
P27	30–39	Mixed White and Black African	Suburban	2	A-levels or equivalent	Yes	Face-to-face
P28	17–29	Any other Asian background	Urban	2	5–7 GCSEs or equivalent	Yes	Telephone, online
P29	40–54	White British	Suburban	4	A-levels or equivalent	Yes	Face-to-face
P30	17–29	White British	Urban	1	<5 GCSEs or equivalent	Yes	Face-to-face, telephone
P31	40–54	White British	Urban	1	<5 GCSEs or equivalent	Yes	Telephone

* 1=most deprived, 5=least deprived.

GCSEGeneral Certificate of Secondary EducationIMDIndex of Multiple DeprivationPparticipant

Our findings are organised into four themes. Differences by age group were apparent and are noted below, as are (fewer) differences by deprivation or educational attainment. Table 3 contains additional quotes for each theme.

**Table 3:** Additional participant quotes for each theme

Theme	Additional quotes
Same pandemic, different impacts	**Ease of accessing methods that do not require an appointment***“…they said we’re sending* [condoms] *to your door. It’s very convenient. They can keep sending this instead of people having to go themselves and use it. It’s very useful and it’s working and that is why they’re doing it and I’m very happy with that.”* [P17]
	**Delays around contraceptive injections***“…it was very difficult to get in-person doctor’s appointments…so I ended up being about a month late on my injection…it was a bit of a stressful period because at the time me and my partner we were living together.”* [P30]
	**Extra support needed when accessing contraceptive services for the first time***“…it was my first time…so for me it was something very important. But let’s imagine for them, probably something that a lot of women ask for…it’s quite…a routine thing they do…’cause I mean it was low priority…but for me that I wasn’t really knowledgeable about talking… I wanted to speak to somebody…”* [P4]
Barriers to accessing contraception	**Challenges of making an appointment***“…its been so difficult to get hold of a doctor…the GP is like an illusion…”* [P22]*“…I had quite a few issues just getting hold of a doctor, just getting a contraceptive full stop.”* [P31]
	**Complex processes that are tricky to navigate***“…it was a…stressful situation…If I could, I would go through…a process which was…smoother…because I went from GP to…the nurse…there was so many…steps towards it.”* [P16]
	**Access to preferred contraceptive method was not treated as a priority***“I was made to feel it was unessential…I remember a couple of times when I was on the pill…it hadn’t arrived because the doctor hadn’t done my prescription…I had to ring up…and say…this is something urgent that I need because obviously I could get pregnant and they were saying well for now you could just use alternative methods of contraception…“* [P20]
	**Diminished quality of appointments***“It was a rushed appointment…there is so much you can’t explain and get into details about symptoms, side effects…“* [P9]
Mixed attitudes towards remote services	**Telephone appointments are problematic for those in school***“At the time when I started* [taking contraception] *I was in school so I was leaving lessons to call my doctor to talk about this. I’m just like in the middle of a school corridor…“* [P15]
	**Online services are quick and easy, especially for younger women***“…it's actually been quite easy…my GP switched everything to EConsult and…as a fairly young person I think an online chat suits me…“* [P28]
	**Anonymity works well but online services can be impersonal***“…even though the online services are anonymous and fast, there’s not a lot of…advice catered towards you in that situation…“* [P3]
	**Remote methods cannot replicate the subtleties of face-to-face appointments***“I just wanna have that interaction of me being able to ask questions…raise doubts…“* [P4]*“…if you’re on contraception that isn’t working, or you’re struggling with it, I don’t think you can really grasp…the affect that they can have over the phone…“* [P24]*“…they will pick up cues from you if you felt uncertain…or that you wanted to say more…“* [P25]
Short-lived versus lasting effects of the pandemic	**Noticing a return to ‘normality’***“Since…we’ve…hit the normality period it’s been really easy and really simple. Obviously now it’s easier to get to a GP, it’s easier to get appointments, but yeah now it’s kind of gone back to how it usually was before.“* [P30]
	**Lasting changes***“I’d never gone back to the Sexual Health Clinic, I’d never gone back to that clinic for this reason, I’ve always stayed with my doctor now…I’m sure it’s open again now but I just don’t access that service any more*.“ [P31]

GPgeneral practitionerPparticipant

### Same pandemic, different impacts

The pandemic impacted participants’ access to contraception unequally, depending on the contraceptive method they were using. Women using barrier methods reported little change or marked improvements in accessibility due to the delivery of free condoms from the SHC. Similarly, those who accessed contraception at the pharmacy described their experiences as “*quick*” and “*straightforward*” when compared with the GP.

*“…because mine was just a case of having a prescription, it was pretty easy…”* [P1]

Those seeking a contraceptive intrauterine device (IUD) experienced issues both at GPs, where fittings were no longer offered, and SHCs, which women often reported as closed. Lengthy waiting times for fittings were reported, causing some to seek out alternative contraceptive methods.

The greatest challenges were faced by those using contraceptive injections (all living in the most deprived areas) prior to March 2020, as they recalled the “*stress*” of being unable to book GP appointments while receiving no signposting to pharmacy provision of injections, experiencing issues with pharmacy provision (discontinuation of products), or feeling uncomfortable about injecting themselves. For some, this resulted in them being unprotected from pregnancy, stopping contraception altogether, or switching to less preferred methods:

*“…it got harder in lockdown because…they shut all the practices…everyone who was having the Depo…they got a phone call saying…‘you can’t come in…look at different alternatives to contraception’, so then I went on the pill.”* [P9]

The pandemic also impacted participants unequally at different life stages. Impacts were greater for women accessing contraception for the first time as they recalled how “*busy*” staff had been and how they felt they had missed out on a “*proper consultation*”. Similarly, those nearing the end of their reproductive lifespan felt “*lost*” regarding their contraceptive needs. As one woman highlighted, the lack of meaningful discussions with her doctor due to COVID-19 restrictions added to feelings of invisibility during this life stage:

*“…I’ve slipped off the scale…I’m…invisible…at my stage…there’s very little…focus on women at my age…towards the end of their fer-fertility…mixed in with the pandemic…”* [P2]

### Barriers to accessing contraception

Women frequently reported barriers to accessing contraception particularly for methods which required a face-to-face consultation. Many felt that contraceptive services were “*inaccessible*” during the pandemic due to service closures, a lack of appointments, “*annoying*” waiting times, and “*unclear*” or “*difficult*” booking processes.

*“…it was impossible to get an appointment in any of the* [sexual health]* clinics…their online service is just so difficult to use…and then when you finally get there they’re booked up for weeks.”* [P27]

Multiple failed attempts to get an appointment, coupled with warnings in the media about the overburdening of the health service during the pandemic, caused one woman to stop using contraception altogether:

*“I think there was…scaremongering as well…I did try, I tried for quite a…few times, but then I…just gave up on it…”* [P6]

Women also reported a lack of communication from service providers on where or how to access contraception if they were unable to provide the service themselves due to COVID-19 restrictions. One woman recalls her GP’s response:

*“Basically,‘that’s it, we’re not giving* [contraceptive injections] *here. You can give it to yourself…that’s it, bottom line’, no other information, or ring this number, or go to this clinic…do it yourself or forget it.”* [P23]

If signposting to other services did occur, it was often vague or unclear. One young woman was signposted to the National Health Service (NHS) website, but this approach fell short of her expectations:

*“I was hoping that I would get an appointment to discuss all the…options for contraception…I was just sent a link to the NHS, basically what I had already read before…it wasn’t very helpful at all…”* [P5]

For those who did get appointments, consultations were often described as “rushed”, which left some feeling unheard or that their contraceptive needs were “*unimportant*” in the context of the pandemic.

*“…you could see that they…didn’t really want to book me in…they didn’t think I needed to be there; they were writing it off…”* [P1]

### Mixed attitudes towards remote services

Participants expressed a range of attitudes when asked about the shift towards remote delivery of contraceptive services. Some stressed that their preferences would be based on what their needs were at the time and that accessing contraception via face-to-face, telephone or online approaches had benefits and drawbacks. Others noted that preferences may change with age and experience. For example, one woman described how a face-to-face appointment was important for new users:

*“If it’s my first-time taking contraception…I wanna ask questions, I wanna know all the possible…side effects, which one would be best for me…I don’t think I will be able to do…an online thing…”* [P4]

Since March 2020, telephone appointments had become more common, and some participants described them as “*easier*” and more “*convenient*” as they saved time and travel, fitted in with schedules, and removed unnecessary trips to the GP or SHC.

However, others found telephone appointments “*difficult*” or “*awkward*”, especially if the calls were unscheduled. This was a particular issue for those living with parents or attending school. One woman noted why impromptu callbacks added to confidentiality concerns:

*“…even speaking on the phone when the doctor does ring back, they don’t always ring back on time, you might be driving, you might be standing in the middle of the supermarket. Whereas a face-to-face appointment…you’ve got that15 minutesinside a room, confidential, you can speak.”* [P23]

Some positive experiences of online access were reported. Ordering prescriptions and communicating with healthcare professionals online facilitated contraceptive access during work hours and removed in-person waiting times. Those who had used online contraceptive services since March 2020 expressed how “*quick*” and “*accessible*” they were, and younger women favoured the anonymity they offered.

*“I…feel more comfortable getting it remotely…there is no judgement. It’s easier, you don’t have to go out of your way to do it…”* [P10]

However, these women also recognised the lack of human interaction as a drawback to using online services.

*“You can misinterpret it, or…it’s hard to convey what you are…trying to say.”* [P10]

Many felt their concerns would be taken “*more seriously*” in person, when practitioners could pick up on visual cues that may be missed during a remote consultation.

### Short-lived versus lasting effects of the pandemic

While some of the women suggested that normal service had resumed, others perceived lasting effects of the pandemic on their experiences of accessing contraception.

*“…things are pretty much back to normal now. I’ve had a few* [contraceptive]* jabs since lockdown…if you tell them what it’s for then they pretty much get you…within a week…”* [P23]

*“…we’re still living with the effects…the restrictions within the GP surgery still seem to remain…they started, during the pandemic…with phone appointments…that hasn’t gone away…is that the pandemic or is that just how the system works* [now]*? …”* [P2]

The replacement of face-to-face consultations with online or telephone appointments described as “*rushed*” and “*impersonal*” was a concern for some of the women. One explained why these lasting effects may impact on the quality of the contraceptive service she receives:

*“…before lockdown I always remember going into the doctor and having anin-depth conversation…since COVID it has just been always over the phone, a quick5-minutecall…that’s it.”* [P20]

The effects of COVID-19 restrictions on accessibility and availability of contraception had left some women feeling their contraceptive needs were viewed as “*unessential*” and not a priority by service providers. These sentiments were exacerbated by wider societal issues around women’s health that contributed to the dismissal of their contraceptive needs as “*optional*”.

Reflecting on her experience, one woman expressed concern about whether future lockdowns would result in similar outcomes:

*“Because it’s happened to us once there’s always in the back of your mind what if there’s a*[nother]*lockdown…you’ll be back to square one…I didn’t expect something like* [contraceptive injections] *to be stopped…”* [P23]

## Discussion

This study utilised qualitative methods to elicit in-depth perspectives on women’s experiences of accessing contraception during the pandemic to complement quantitative data presented in the literature.^57 9 13 14 16^,^18^ We found inequalities in women’s experiences of accessing contraceptive services since March 2020, lasting effects that continue to create barriers to access, and varying views on the suitability of remote methods of service delivery.

Quantitative studies have indicated that COVID-19 prompted service closures,^5^ face-to-face capacity restrictions,^5 16^ and access difficulties,^916^,^18^ particularly for those seeking LARC.^7^ Our participants generally reported the same issues, but elaborated on the real-world impacts of inaccessible services and highlighted the mental and physical implications of an unmet need for contraception during this time. Similar to work from the early stages of the pandemic,^15 16 18^ we also found complex booking systems, reduced appointment availability, a lack of information or signposting from services, and reduced quality of appointments as barriers to access. Our findings indicate some of these barriers persisted beyond 2020, with lasting effects still reported in late 2022.

Previous research has suggested self-censorship of need and hesitation towards accessing contraceptive services was present during COVID-19.^15 18^ Our participants also recalled feelings of uncertainty regarding the importance of asserting their contraceptive needs during the pandemic.

Like other studies,^13^,^16^ many of our participants had embraced the transition towards more remote methods of service delivery and praised the ease and convenience with which they could access contraception during the pandemic. However, our participants also recognised limitations to accessing contraception this way and face-to-face appointments remained the preferred mode of access for asking questions or expressing concerns. Confidentiality was a key concern; discussing contraception over the phone was particularly difficult for those attending school or living with parents, supporting earlier work by Bosó Pérez *et al.*^15^

Beyond existing research, our findings shed light on the unequal impacts felt by women, depending on their preferred contraceptive method. The greatest barriers were reported by those using contraceptive injections prior to March 2020, resulting in method switching or stopping contraception altogether. We also found that women accessing contraception for the first time or those nearing the end of their reproductive lives were less likely to feel their needs had been met as they felt unable to access adequate support or guidance. This suggests women would like more opportunities for in-depth discussions with practitioners at these life stages. One option would be to provide contraceptive counselling, to help women at different stages of their reproductive lives by encouraging shared decision-making between patient and practitioner and integrating individual needs into contraceptive choice.^24^ Research shows that contraceptive counselling improves contraceptive continuation,^25^ increases satisfaction with current contraceptive method,^26 27^ and promotes LARC uptake.^28^ However, the effectiveness of digital approaches to contraceptive counselling will need to be considered if remote delivery of contraceptive services continues in England.

### Strengths and limitations

Our sample is diverse in terms of age, ethnicity, contraceptive methods used, services accessed, and mode of access. However, findings may not apply to wider populations of women in England, the UK, or elsewhere. We were unable to recruit any participants living in the East of England or North East where service provision and experiences may have differed. The sample was purposely skewed towards women of lower educational attainment and IMD1. We found relatively few differences according to these criteria. Using educational level on its own may not be an adequate indicator of lower social grade, especially when speaking with younger women who have not yet finished school. It may be beneficial to recruit women according to poverty-based factors, such as receipt of benefits, low paid work, reliance on public transport, or caring responsibilities. All our participants were digitally recruited via a market research agency. Future research is needed to capture the first-hand experiences of non-internet users or women whose first language is not English as they are likely to have faced additional barriers to access. Most of the women in our sample used contraception to prevent pregnancy. We have not included women who primarily used contraception to control symptoms of menstrual conditions, and the perceived importance of their contraceptive needs during COVID-19 may differ from those in our study. Finally, it is difficult to ascertain whether some of the issues the women experienced were a direct result of the pandemic, as funding cuts to sexual health services and transitions to more remote methods were already underway in England.^29^ While the pandemic accelerated many of these barriers, other factors may also be responsible.

## Conclusions and recommendations

The experiences of our participants highlight the pervasiveness of short-term and lasting impacts of the pandemic on contraceptive services in England. Our findings suggest more steps are needed to ensure appropriate ease of contraceptive access in future outbreaks and to overcome prevailing barriers around LARC availability and reduced quality consultations. Future qualitative work with healthcare professionals would offer an alternative perspective on the perceived importance of delivering contraceptive services during the pandemic. Based on our findings, we have developed recommendations for policymakers that are designed to maximise uptake and improve user experience given the shifting landscape of contraceptive services in England (table 4). The recommendations are divided into two areas of focus: access to services and service delivery. They also take into consideration the adoption of a life-course approach and the move towards an integrated model of women’s health services as outlined in the Women’s Health Strategy for England.^30^

**Table 4:** Recommendations for policymakers

Focus area	Recommendation
Access to services	Increase the availability of appointments for contraception, especially long-acting reversible contraception (LARC) procedures, and have systems in place to ensure women are signposted to a nearby service where they can get a LARC appointment if it is not available or there are long waiting times at their current point of access.
	Review booking systems to make them more user-friendly and create more access points to address barriers associated with lengthy waiting times when booking appointments by telephone or attending drop-in centres.
	Clarify post-pandemic processes on how and where to access contraception to alleviate any confusion caused by COVID-19-related policy and practice changes.
	Ensure remote systems and consultations that were introduced during the pandemic remain available for women who favoured the ease and convenience of these modes of access, and strive to make these more user-friendly to encourage more women to switch to accessing contraception this way when appropriate.
Service delivery	Invest in education and training for health practitioners to identify significant time points and life stages when women would benefit from increased contraceptive support and improved interactions at these time points, for instance, when accessing contraception for the first time or during perimenopause.
	Contraceptive services should offer additional support or contraceptive counselling to those who accessed contraception for the first time during the pandemic as restrictions around policy and practice most likely impacted their experience of accessing contraception and they may be unsatisfied with their current contraceptive method.
	Contraceptive services should continue to provide in-person consultations for women who prefer to access contraception this way. Choice is valuable and it is important that women have the option to receive in-person support when they feel they would benefit from this.

## Data Availability

No data are available.
